# Effects of education on self-monitoring of blood pressure based on BASNEF model in hypertensive patients

**Published:** 2010

**Authors:** Mohammad Hossein Baghianimoghadam, Zohreh Rahaee, Mohammad Ali Morowatisharifabad, Gholamreza Sharifirad, Abas Andishmand, Leila Azadbakht

**Affiliations:** a Associate Professor in Health Education, School of Health, Shahid Sadoughi University of Medical Sciences, Yazd, Iran; b MS in Health Education, School of Health, Shahid Sadoughi University of Medical Sciences, Yazd, Iran; c Assistant Professor in Health Education, School of Health, Shahid Sadoughi University of Medical Sciences, Yazd, Iran; d Associate Professor in Health Education, School of Health, Isfahan University of Medical Sciences, Isfahan, Iran; e Assistant Professor in Cardiology, School of Medicine, Shahid Sadoughi University of Medical Sciences, Yazd, Iran; f Assistant Professor in Nutrition, Nutrition and Food Security Research Center, School of Health, Isfahan University of Medical Sciences, Isfahan, Iran

**Keywords:** Education, Self-monitoring, Hypertension, BASNEF Model

## Abstract

**BACKGROUND:**

Hypertension is one of the most important health problems. Self-monitoring may be an effective method for controlling this disease. The aim of this study is to determine the effectiveness of education on blood pressure self-monitoring in patients with hypertension based on BASNEF model.

**METHODS:**

In this clinical trial, 150 outpatients with hypertension were randomly selected from those referred to private clinics in Yazd, Iran, during 2008. They were divided in two groups. The data were collected by a validated and reliable questionnaire. The implementing educational program was continued for 2 months and the pre-test and post-test had an interval of 2-months. BASNEF model was applied to explain the motivation of a behavior. Descriptive analysis, correlation test and also regression analysis were used to analyze data.

**RESULTS:**

The respondents acquired 17.72% of total score for self-monitoring behavior, 47.03% of attitude, 12.37% of subjective norms, 33.46% of intention and 50.95% of enabling factors. After intervention, there were significant increases in self-monitoring behavior (173.31%), attitude (62.60%), subjective norms (54.70%), intention (129.93%) and enabling factors (46.62%) in the intervention group. There was no significant difference between the constructs of BASNEF model in the control group after intervention compared to the baseline values (p > 0.05).

**CONCLUSIONS:**

The results of this study showed that the level of self-monitoring behavior in the patients was low. Educational programs are helpful and necessary to improve self-monitoring behavior in patients with hypertension.

Hypertension is one of the public health problems in the world,^1^ which afflicts about 28% of North Americans, 44% of Europeans and 26% of Eastern Mediterraneans aged 35 to 64 years.^2^ In Iran, it afflicts about 26.6% of over 15-year-old population.^3^ Hypertension is the most common of all cardiovascular diseases, often leading to stroke, heart attack, kidney disease and aortic aneurysm.^4^ The etiology of hypertension is relatively unknown. However, several factors including obesity,^5^ diet^6^–^8^ and physical activity have been identified as possible factors. Hypertension as a primary contributor to disability and mortality usually cannot be cured, but must be managed. Medication is the leading therapy for treating hypertension. More than one hundred different medications are available for the treatment; all have proven efficacy, most have few side effect, and many are formulated for once-daily dosing. The reported rates of blood pressure control are so disappointing. The success rate for hypertension control in the United States is only 27%.^9^ The level of awareness of this disease in the Asian cities such as in Delhi is 50%^1^ and the treatment rate of the hypertension among elderly Asians is 25%.^10^

The majority of hypertensive patients do not reach the current target blood pressure (BP) levels set by the international guidelines.^11^ Physicians have traditionally give advice on what to do to avoid dreaded health complications in the future.^12^–^14^ The most challenging issues in education involve helping patients to make long-term lifestyle change.^12^ The way in which education is implemented can be important in helping patients to make long-term lifestyle changes.^15^

Self-measurement of BP or home BP measurement is an important way for controlling this disorder.^16^ In this low-cost method, a high frequency of measurement and a possibility to evaluate the circadian alternation of BP is simply available.^17^,^18^ Home measurements abolish the discrepancy created by white coat hypertension^17^ and improve pharmacologic compliance.^19^ There are conflicting results from different studies regarding the effects of home blood pressure measurements.^20^,^21^ A recent clinical trial on the Electronic Communications and Home Blood Pressure Monitoring revealed that self-monitoring blood pressure could improve the care of large numbers of patients with uncontrolled hypertension.^22^ A research in this regard, also indicated that home blood pressure was superior to office BP in hypertensive patients.^23^

There are some educational models in health education field, which have been used for determining correlates of healthy behaviors.^24^ The Beliefs, Attitude, Subjective Norms, Enabling Factors (BASNEF) model is one of the health education models.^25^ Effective health education requires an understanding of the factors which underlie a person’s behavior.

BASNEF is a simplified approach to understanding behavior, which is based on the PRECEDE model and on Value Expectancy Theory. Applying the BASNEF approach requires determining community perspectives regarding the behavior. The behavior should be defined precisely. Paying attention to the facilities and knowledge required for a motivated person is also important. So, all the enabling factors for a motivated person should be available.^26^ Therefore, by understanding the community, BASNEF model can be used to design health education intervention.

Education on self-monitoring of blood pressure has not been emphasized in Iran. Previous worldwide studies on this topic have not been based on an educational model.^11^,^16^,^22^ The health education studies are needed to apply in different populations; therefore, the aim of this study is to determine the effectiveness of education on self-monitoring of blood pressure in patients with hypertension based on BASNEF model.

## Methods

This clinical trial was carried out during the year 2008 in Yazd, Islamic Republic of Iran. Patients were randomly selected from private clinics. A patient met the inclusion criteria if he/she had at list one year essential hypertension. This was defined either by a history of antihypertensive medication treatment or a mean BP value of 140/90 mm Hg or higher, which was measured twice each time at four consecutive occasions. All patients consumed similar medications during the study.

Elderly patients who could not understand the questions were excluded from the study. The study population consisted of 150 patients. The samples were randomly divided in two groups (75 in case group and 75 in control group). For data collection, the author interviewed the outpatients who referred to private clinics. There was no difference between the two groups in age, sex, marital status, education, job, body mass index and smoking. The BP readings were similar in the two groups at the beginning of the study. The researcher was the educator and instructed the patients on how to measure BP at home. Each educational session was 30 to 40 minutes. During educational sessions, teaching methods such as group discussion, question and answer using pamphlets, CD and Power-Point were used based on the BASNEF model. The place of education was the meeting room of the private clinics. The educational content of each session involved the prevalence of hypertension, mortality rate from the hypertension, importance of the self-monitoring BP, how to measuring BP at home, how to provide a sphygmoma-nometer for patients, the role of family in helping the patients, lower cost of BP monitoring versus the higher costs of the treatment, and some recommendations for lowering the BP. All patients had signed the written consent form. This study was confirmed by the research council and ethical committee of the Shahid Sadoughi University of Medical Sciences, Yazd.

The questionnaire was designed by the researchers, but the scoring method was based on the Likert scales. The questionnaire had three sections: demographic data (6 questions), medication data (11 questions), BASNEF model (57 questions) including 18 questions on attitude, 12 questions on social norms, 7 questions on BP, 8 questions on enabling factors and 12 questions on self-monitoring. Attitude questions consisted of two sections: 9 questions on belief and 9 questions on evaluation belief. Answers were based on 5 Likert scales (certainly agree = 4, agree = 3, no idea = 2, disagree = 1 and certainly disagree = 0) and the score range for every section was 0 to 36 and the total score of attitude was a multiple of scores for the two sections. The 12 questions related to social norms were also divided into two sections: 8 questions on normative beliefs and 4 questions on motivation for complying. The answers were based on 5 Likert scales and the total scores of social norms was a multiple of scores for the two sections. The range of score for social norms was 0 to 512. BP was measured with 7 questions and its score range was 0 to 28. Enabling factors were measured by 8 questions with 4 Likert scales answers and the score range was 0 to 24. Finally, 12 questions on self-monitoring were based on 5 Likert scales with minimum 0 and maximum 48 scores.

The case group performed a double BP measurement twice daily (in the morning and evening). In the control group, the measurements were made only in the beginning and at the end of the study. Furthermore, regular office measurements were made at the beginning and at the end of the study in both groups.

The patients kept a dairy on their BP values and returned it to the researcher after 2 months. Demographic data, data based on the BASNEF model and medication data were collected both at baseline and at the end of the study.

To ensure the clarity of questionnaires, pilot testing of the questionnaire was performed using the coherence and consistency upon 20 participants who were not included in the survey.

After that, the questionnaire was modified based on participants’ feedback. Content validity was established by 5 experts who were academic staff and health educators. So, content validity of the questionnaire was assessed through view points of an expert panel. The reliability of questionnaire was determined by Cronbach’s alpha, which was in the range of 0.66 to 0.87.

Comparison between two groups was done by Wilcoxon test. Pearson correlation analysis was used for determining the correlation between variables of BASNEF model. Regression analysis was used for determining power of the model in predicting BP self-monitoring. P value less than 0.05 was considered as significant level.

## Results

A total of 150 patients entered the study. About 39% of the participants were male and 61% were female. The mean age was 57.9 ± 9.9 years with a range of 36-70. The education level of 22% was diploma or precollege degree, 6.7% went to high school and guidance school, 42.7% just went to primary school and 28.7% were illiterates. Of all, 56.7% of the participants were housewives, 6.7% were government employees, 16% were working in private sector and 20.7% were retired. The mean history of hypertension was 6.77 ± 7.7 years; 90.6% reported adherence to anti-hypertensive medications.

As presented in table 1, there was a significant difference between the mean scores of all of the constructs of BASNEF model in case group after intervention compared to the baseline values (p < 0.001). However, there was no significant difference between the constructs of BASNEF model in control group after intervention compared to the baseline values (p > 0.05). The mean scores of intention for controlling the BP in case and control groups were very low (9.62 and 9.12 out of 28, respectively) at baseline, and in the case group increased to 22.12 after the intervention. However, it decreased to 8.48 in control group at the end of the study. The mean scores of self-monitoring of the two groups were very low (8.92 and 7.74 out of 48, respectively) at baseline, which increased to 24.38 in the case group and decreased to 7.45 in the control group at the end of the study.

**Table 1:** Mean ± SD of grades of constructs of BASNEF model in participants before and after intervention

Variables	Groups	Before intervention Mean ± SD	After intervention Mean ± SD	P value
Attitude	Case	608.4 ± 297.47	989.28 ± 254.74	0.001
	Control	610.72 ± 329.49	613.38 ± 327.08	0.317
Subjective norms	Case	44.29 ± 82.38	68.52 ± 84.7	0.001
	Control	82.38 ± 122.55	82.38 ± 122.55	1
Intention	Case	9.62 ± 8.63	22.12 ± 10.49	0.001
	Control	9.12 ± 8.87	8.48 ± 9.16	0.255
Enabling factor	Case	13.77 ± 5.14	20.19 ± 2.1	0.001
	Control	10.69 ± 5.73	10.8 ± 5.79	0.102
Self-monitoring	Case	8.92 ± 9.83	24.38 ± 19.93	0.001
	Control	7.74 ± 10.05	7.45 ± 10.67	0.861

Table 2 shows the correlation coefficients among variables of BASNEF model. There were a positive significant correlation between attitude and subjective norms and intention for controlling BP (p < 0.01). This was the same regarding subjective norms, intention for controlling BP, enabling factors and self-monitoring (p < 0.01). There were positive correlations among the intention and enabling factors and self-monitoring (p < 0.01). Also, there was a positive correlation between enabling factors and self-monitoring. But there was no significant correlation among attitude and enabling factors and self-monitoring (p > 0.05).

**Table 2:** Correlation coefficients among variables of BASNEF model

Constructs of BASNEF model	Attitude	Subjective norms	Intention	Enabling factors
Attitude	1	-	-	-
Subjective norms	0.272 *	1	-	-
Intention	0.217 *	0.565*	1	-
Enabling factors	−0.009	0.334 *	0.587 *	1
Self-monitoring	−0.055	0.402 *	0.677 *	0.735 *

* p < 0.01

Data presented in table 3 shows that the explain variance in intention for self-monitoring was 23% and behavior for self-monitoring was 58%.

**Table 3:** Rates of analyses regression of constructs of BASNEF model

Independent variables	β standard	P value	R^2^	Dependent variable
Attitude	0.078	0.296	0.226	Intention for self-monitoring
Subjective norms	0.450	0.001		
Intention for self-monitoring	0.463	0.001	0.577	Behavior for self-monitoring
Enabling factors	0.393	0.001		

Regarding the role of demographic variables, women had better attitude compared to men (p < 0.001). Higher education level was associated with higher intention for controlling BP and increasing enabling factors for self-monitoring (p < 0.001). There were no significant correlation between other demographic variables and the constructs of BASNEF model.

## Discussion

The results of the present study revealed that educational intervention based on BASNEF model improves hypertensive patients’ self-monitoring abilities. The behavioral intention and intention for measuring BP in the case group improved significantly after intervention. This might be related to the change that intervention program made in participants’ subjective norms and enabling factors. According to the previous studies, enabling factors could affect the attitude changes.^26^–^30^ In the mentioned studies, scores of enabling factors, which were among main components of the BASNEF educational model, were significantly improved after intervention in the case group. It could be concluded that these factors provide information and lead to intention of behavioral action for BP self-monitoring. In contrast, these factors were not changed in the control group in the present study and subsequently patients’ attitude and intention for BP monitoring did not changed. Use of enabling factors improved some preventive behaviors in the case group as mentioned in another study.^31^ It seems that enabling factors have an important role in changing intention of mindful behavior.

In the present study, the subjective norms interference measured after the intervention in order to determine the role of subjective norm on the participants’ behavior in monitoring their BP. This is one of the most important strengths of this study. However, some health education studies measured social norms before intervention in order to determine the influence of the target group’s subjective norms for designing educational action without evaluating the level of effectiveness after intervention.^30^,^31^

In the present study, the mean scores of constructs of BASNEF model increased in the case group after intervention, but it decreased or was the same in the control group. These results support the findings of many other studies conducted on these variables.^29^–^34^ The present findings revealed a significant correlation between self-monitoring behavior and intention for BP control and enabling factors. These results support the results of Lee,^28^ who showed a significant correlation between intention and behavior of self-management of BP. Rhodes^35^ demonstrated a strong significant correlation between intention and behavior. Down and Hausenblas^36^ showed a strong correlation between intention and behavior in their study. In this study, there was a significant correlation between the attitude and intention and subjective norms. These correlations show the efficacy of BASNEF model in prevention and control of chronic disease. There was a significant correlation between all constructs of BASNEF model except attitude with enabling factors and self-monitoring, that was in line with the results of other studies.^28^,^30^,^37^

Besides the effectiveness of the BASNEF model in health education studies, other educational models such as PERECEDE or health belief model have important roles in increasing the knowledge, attitude and behavior of target groups related to health and disease.^38^,^39^ Several researches mentioned the beneficiary of applying different models.^38^–^42^ In a study on type 2 diabetic patients, nutritional education based on health belief model could reduce fasting blood sugar significantly compared to a control group.^42^ Although nutrition education increased the score of knowledge among this group, choosing an appropriate educational model helped to increase the score of behavior and also changed the metabolic variables.^42^

Using an educational model for increasing the behavior scores for self-monitoring blood pressure is a positive point in the present study that can improve the results of the educational intervention. Choosing a suitable educational model in different health educational studies should be considered in interpretation of the results. However, more time is needed to see the effect of educational intervention on the level of BP. So, just the change in behavior after the intervention and the measurement of the BP was considered and its probable change was not the aim of this short-term study, which could be considered as a limitation for the present research.

In the present study, women had better attitude and were more successful regarding the self-measurement of the blood pressure compared to men. This might be due to the higher level of sensitivity and health consciousness in women, as Cutler et al also indicated in their study.^43^ Higher educational level was associated with higher intention for controlling the BP and increasing enabling factors for self-monitoring of BP in the present study, which was in the same line with Vitezic et al results.^44^ Education on self-monitoring BP for educated patients is more successful in changing their behavior compared to not educated ones. Patients with higher education level also can use sphygmomanometers more precisely. Physicians and health care providers should advise the patients to check the accuracy of their home sphygmomanometers regularly.^45^ There were no significant association among other demographic variables and the constructs of the BASNEF model in the current study. However, socioeconomic status of the patients was an important demographic variable correlated with the self-monitoring BP in African American adolescents,^46^ which should be considered in the health educational studies.

## Conclusions

Increasing self-monitoring BP among hypertensive patients is largely recommended.

Considering the enabling factors as the most important factors of self-monitoring in hypertensive patients, securing the sphygmomanometer for patients (preferably free) is necessary. Planning educational programs based on self-monitoring of hypertension is necessary too.

Subjective norms can influence on self-monitoring behavior by means of enabling factors and self monitoring intension. Moreover, we believe that strategies to involve family members, relatives or friends who have influence on patients in intervention programs, specially confirmation of them by physician or other health care personnel can strongly effect self-monitoring of patients especially females.

BASNEF model is a powerful model in forecasting self-monitoring behavior and can be a base for educational intervention. Thus, for improving self-monitoring behavior and finally controlling the disease, using such educational programs used in this study is recommended and emphasized.
